# Sustainable Concrete with Waste Tire Rubber and Recycled Steel Fibers: Experimental Insights and Hybrid PINN–CatBoost Prediction

**DOI:** 10.3390/polym17212910

**Published:** 2025-10-30

**Authors:** Ali Serdar Ecemiş, Sadik Alper Yildizel, Alexey N. Beskopylny, Sergey A. Stel’makh, Evgenii M. Shcherban’, Ceyhun Aksoylu, Emrah Madenci, Yasin Onuralp Özkılıç

**Affiliations:** 1 Department of Civil Engineering, Necmettin Erbakan University, Konya 42090, Türkiye; 2 Department of Civil Engineering, Engineering Faculty, Karamanoglu Mehmetbey University, Karaman 70200, Türkiye; 3 Department of Transport Systems, Faculty of Roads and Transport Systems, Don State Technical University, 344003 Rostov-On-Don, Russia; 4 Department of Unique Buildings and Constructions Engineering, Don State Technical University, 344003 Rostov-On-Don, Russia; 5 Department of Engineering Geometry and Computer Graphics, Don State Technical University, 344003 Rostov-On-Don, Russia; 6 Department of Civil Engineering, Konya Technical University, Konya 42250, Türkiye; 7 Department of Technical Sciences, Western Caspian University, Baku 1001, Azerbaijan

**Keywords:** waste tire rubber, recycled tire steel fibers, sustainable concrete, recycled aggregates, physics-informed neural networks

## Abstract

The growing environmental concern over waste tire accumulation necessitates innovative recycling strategies in construction materials. Therefore, this study aims to develop and evaluate sustainable concrete by integrating waste tire rubber (WTR) aggregates of different sizes and recycled waste tire steel fibers (WTSFs), assessing their combined effects on the mechanical and microstructural performance of concrete through experimental and analytical approaches. WTR aggregates, consisting of fine (0–4 mm), small coarse (5–8 mm), and large coarse (11–22 mm) particles, were used at substitution rates of 0–20%; WTSF was used at volumetric dosages of 0–2%, resulting in a total of 40 mixtures. Mechanical performance was evaluated using density and pressure resistance tests, while microstructural properties were assessed using scanning electron microscopy (SEM) and energy-dispersive X-ray spectroscopy (EDS). The findings indicate systematic decreases in density and compressive strength with increasing WTR ratio; the average strength losses were approximately 12%, 20%, and 31% at 5%, 10%, and 20% for WTR substitution, respectively. Among the WTR types, the most negative effect occurred in fine particles (FWTR), while the least negative effect occurred in coarse particles (LCWTR). The addition of WTSF compensated for losses at low/medium dosages (0.5–1.0%) and increased strength by 2–10%. However, high dosages (2.0%) reduced strength by 20–40% due to workability issues, fiber clumping, and void formation. The highest strength was achieved in the 5LCWTR–1WTSF mixture at 36.98 MPa (≈6% increase compared to the reference/control concrete), while the lowest strength was measured at 16.72 MPa in the 20FWTR–2WTSF mixture (≈52% decrease compared to the reference/control). A strong positive correlation was found between density and strength (*r*, Pearson correlation coefficient, ≈0.77). SEM and EDX analyses confirmed the weak matrix–rubber interface and the crack-bridging effect of steel fibers in mixtures containing fine WTR. Additionally, a hybrid prediction model combining physics-informed neural networks (PINNs) and CatBoost, supported by data augmentation strategies, accurately estimated compressive strength. Overall, the results highlight that optimized integration of WTR and WTSF enables sustainable concrete production with acceptable mechanical and microstructural performance.

## 1. Introduction

With the growing awareness of environmental issues, concerns about minimizing environmental damage and recycling waste materials are becoming increasingly important. The construction industry is a multidisciplinary sector where many materials, including raw materials, are used. The use and recycling of waste materials have begun to play an increasingly important role in the construction industry, and numerous studies have been initiated on this subject. As the population grows and transportation needs increase, the amount of waste tires and the environmental problems caused by this waste also increase in parallel. It is estimated that 1.5 billion tires are produced in the world each year. However, it is estimated that approximately 1 billion tires reach the end of their useful life each year, and more than 50% of these are disposed of in landfills or as waste, without undergoing any processing. In 2030, the number of tires from motor vehicles is expected to reach 1200 million, which means that approximately 5000 million tires will be discarded on a regular basis [1,2,3].

Non-biodegradability of waste tires poses a problem for the environment [4,5,6]. For this reason, the use of waste tires instead of aggregates has emerged as a suitable, innovative, and environmentally friendly alternative for the disposal and utilization of tires, which are very difficult to biodegrade in nature; numerous studies have been initiated [7]. In studies examining the properties of concretes produced by adding tire pieces, it has been observed that there is a general decrease in mechanically significant mechanical properties such as tensile and compressive strength [1,8,9,10,11]. This decrease in compressive strength increases with the increasing ratio of tires used and with the increasing size of the tire used [8,12,13]. While rubber negatively affects the mechanical properties of concrete, it has beneficial effects. The results of the studies show that structural applications such as reinforced concrete slabs, columns, beams, and rubberized concrete walls perform better under high-velocity impact, collision, and blast loads compared to conventional concrete. In addition, significant improvements have been observed in the dynamic properties of structural elements under cyclic and seismic loads, including ductility, energy distribution, energy dissipation under impact, and fracture toughness [14,15,16]. Structural materials with high damping properties are effective when used to reduce dynamic impacts from loads such as seismic loads. In the study, the damping coefficient of rubberized concrete increased by 62% compared to normal concrete, and the seismic response acceleration of the structure decreased by 27% [17,18]. Kaewunruen et al. reported that rubber particles can be used to improve splitting tensile and flexural strengths, damping properties, and electrical resistance [19]. Various treatments were applied to the rubber to eliminate the negative effects on the mechanical properties of concrete, with the aim of improving these properties. In the studies, pre-coating and presoaking methods were investigated, and remarkable results were obtained for improving mechanical properties [8,20,21,22].

Another product that can be obtained or recycled from waste tires is the steel fibers contained within them. In the literature, there are studies on concrete produced by adding industrial steel fibers, other fiber types, and the effect of these fibers on concrete behavior [23,24,25]. Studies have shown that steel wires cause a significant increase in some mechanical properties of concrete such as flexural and tensile strength [26,27]; on the other hand, the effect on the compressive strength was not very significant [24,28,29]. However, to achieve effective strength increases, it is necessary to carry out a careful evaluation of parameters such as aspect ratio, shape, surface properties, quality, and fiber content for optimal results [30]. In addition, the bridging effect of steel fibers reduces the negative effect caused by the use of rubber on flexural and shear strength [31,32]. Studies comparing the behavior of concrete produced using waste tire fibers and industrial fibers have shown similar results [25,33,34,35]. The present study examined the dynamic pressure behavior of concrete produced using wire obtained from waste tires. The optimum fiber dosage in terms of workability and compressive strength was determined to be 0.75 units [36]. In the study conducted by Zia et al., hybrid SFRC produced using fibers obtained from both industrial and waste tires was examined. Increases in mechanical strength properties were achieved compared to normal concrete [37].

Another area of research to improve the mechanical properties of WTRC concrete involves the combined use of waste rubber and fibers to benefit from the positive effects. In the study by Peng et al., the highest compressive strength was achieved with the addition of 1% steel fiber, and the maximum tensile and flexural strengths were achieved with the addition of 1.5% steel fiber. The best mechanical strength enhancement and the best toughness of WT-RSFC were obtained when using 10% rubber and 1.5% scrap steel fiber [38]. Dong et al. obtained high compressive strength (49 MPa–56 MPa) in concrete produced using 1.5% end-hooked steel fiber and 20% rubber fragments obtained from waste tires. They stated that it can be used in structural engineering. Formulas for compressive strength, elastic modulus, and the peak strain of optimized WT-RSFC were proposed [39]. In a study examining the shear behavior of reinforced concrete beams produced using steel fiber and crumb rubber, an increase in strength, moment capacity, and ductility was achieved when rubber was used in excess of 10% by volume in combination with steel wire [40]. Ul Islam et al. reported that the use of fibers in hybrid form gives better results [41].

The novelty of this study lies in the integration of an experimental and analytical framework that simultaneously investigates the mechanical and microstructural performance of concrete integrated with waste tire rubber (WTR) aggregates and recycled steel fibers (WTSF), while introducing a hybrid physics-informed neural network (PINN)–CatBoost model for predictive analysis. Unlike conventional empirical or purely data-driven models, the proposed hybrid approach embeds the physical constraints of concrete behavior into the neural network architecture. This integration ensures physically consistent predictions despite the limited experimental data, thereby addressing one of the major challenges in modeling sustainable concretes. Furthermore, the developed model demonstrates high accuracy and generalization capability compared to standalone PINN and traditional machine learning algorithms, offering a pioneering digital tool for optimizing rubberized concrete in sustainable construction applications.

## 2. Materials and Methods

### 2.1. Details of Materials

**Figure 1:**
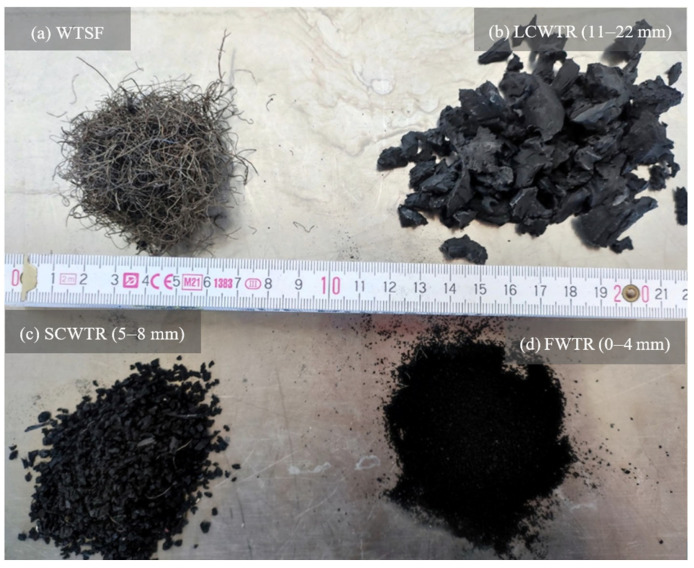
Waste materials used in the concrete: (**a**) Recycled waste tire steel fibers (WTSF); (**b**) Large coarse waste tire rubber (LCWTR); (**c**) Small coarse waste tire rubber (SCWTR); (**d**) Fine waste tire rubber (FWTR).

In this study, waste tires of three different particle size ranges were incorporated into concrete mixtures. The waste tire rubber (WTR) particles were sieved to specified gradations and used as-received; no chemical or mechanical pre-treatment (e.g., alkaline washing, abrasion, etc.) and no oven-drying step were applied. Rubber particles sized from 0 to 4 mm (Fine Waste Tire Rubber, FWTR), from 5 to 8 mm (Small Coarse Waste Tire Rubber, SCWTR), and from 11 to 22 mm (Large Coarse Waste Tire Rubber, LCWTR) were added to produce 15 × 15 × 15 cm cube specimens at substitution levels of 0%, 5%, 10%, and 20% for each particle group. Alongside the rubber particles, steel fibers (SF) extracted from end-of-life tires were introduced as an additional reinforcement material. Owing to their elevated carbon content, these recycled fibers possess remarkable strength and durability. The fiber dosages were adjusted to 0%, 0.5%, 1%, and 2%. Both the waste tire rubber (WTR) aggregates and the recycled waste tire steel fibers (WTSF), supplied by regional recycling facilities, are shown in Figure 1. Ordinary Portland cement of type CEM I 32.5 R, provided by Konya Çimento A.Ş. (Konya, Türkiye), served as the binder, while tap water was employed for mixing, and curing the concrete samples. The compressive strength and unit weight of the specimens were determined, scanning electron microscopy (SEM) and energy-dispersive X-ray spectroscopy (EDS) were used for microstructural characterization with Hitachi-SU 1510 instrument (Hitachi, Tokyo, Japan) in Necmettin Erbakan University BİTAM labs in Konya, Türkiye.

### 2.2. Details of Production

All concrete specimens were produced in the Construction Laboratory of Necmettin Erbakan University. The replacement of natural aggregates with WTR was made on a volume basis, considering the near equivalence between their unit weights and volumes (≈1.00 t/m^3^). In other words, because the unit volume weight of the rubber aggregates is close to 1.00 t/m^3^, their weight and volume were considered equivalent during mix calculations. Therefore, the substitution calculations in Table 1 are based on the volume replacement method. Including the control specimens, which do not contain WTR and WTSF, a total of 40 different mixes were prepared. The mix proportions and quantities, calculated using the volume replacement method, are presented in Table 1. Concrete mixtures were labeled based on the type and content of waste tire rubber (WTR) aggregates and waste tire steel fibers (WTSFs). Concrete mixtures were coded as [Rubber content + Rubber type] − [Steel fiber content]. For instance, 0WTR-0WTSF denotes the control mix without WTR and WTSF, 5SCWTR-0WTSF indicates 5% replacement with Small Coarse WTR and no WTSF, 10FWTR-1WTSF corresponds to 10% Fine WTR with 1% WTSF, and 20LCWTR-2WTSF represents 20% Large Coarse WTR with 2% WTSF. Here, the first number (0, 5, 10, 20) refers to the replacement percentage of natural aggregate by WTR, while the second (0, 0.5, 1, 2) specifies the WTSF dosage.

## 3. Hybrid PINN-CatBoost Prediction

**Figure 2:**
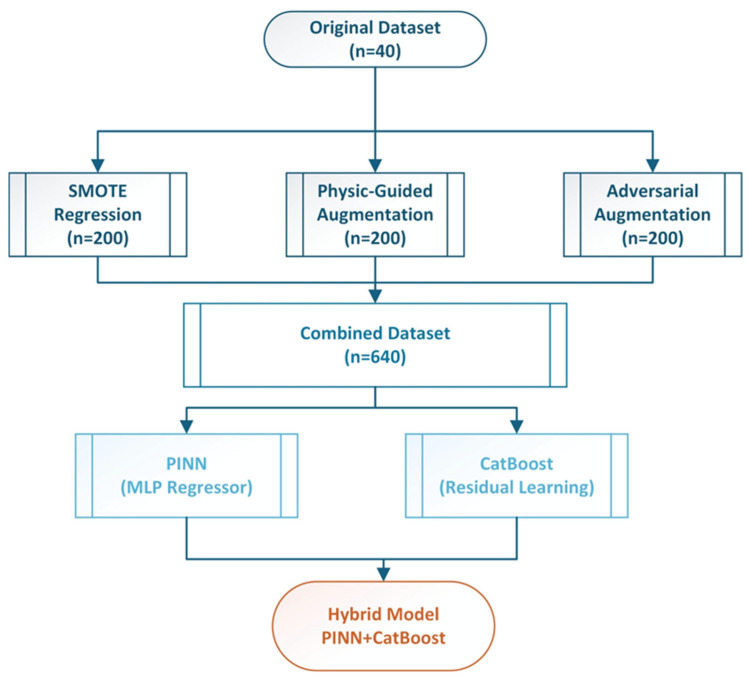
Hybrid PINN-Cat Boost Methodology.

**Figure 3:**
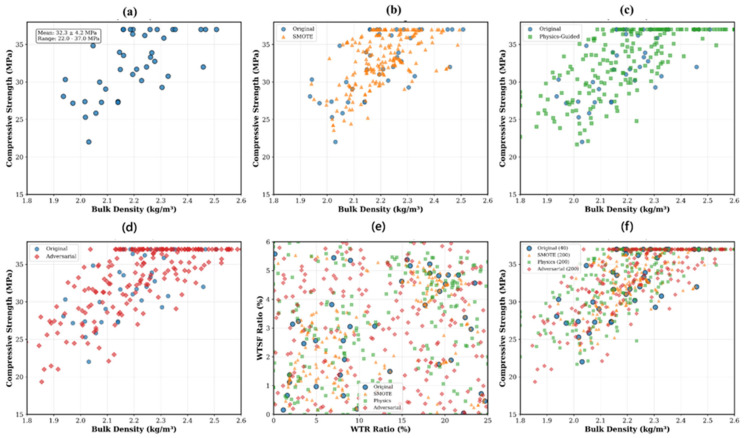
Data augmentation strategies and their effects: (**a**) Original Dataset, (**b**) SMOTE Regression, (**c**) Physic-Guided, (**d**) Adversarial, (**e**) Feature space coverage, (**f**) Combined Dataset (*n* = 640).

The experimental dataset (Table 1) comprised 40 concrete samples reinforced with varying proportions of WTR and WTS. Input parameters were bulk density, *WTR* ratio (%), *WTSF* ratio (%), and WTR type (SC: steel cord; F: fiber; LC: large chip; and none: control). The output consisted of compressive strengths at 28 days with a range between 16.72 and 36.98 MPa. The dataset exhibited a mean of 28.21 ± 5.52 MPa, which represents the typical values for modified concrete composites based on the ingredients. In addition, feature correlation analysis indicated that bulk density had the most significant positive association with compressive strength (*r* = 0.771, *p* < 0.001), while WTR ratio displayed a notable correlation (*r* = −0.598, *p* < 0.001). The WTSF ratio had a minor impact (*r* = 0.088), whereas the WTR type displayed a substantial effect (*r* = 0.303). These correlations informed the proposed physics-guided augmentation strategy and model architecture design. To address the limited dataset, a comprehensive data augmentation approach was established, integrating three separate techniques, to provide synthetic training examples while maintaining fundamental physical correlations. The flowchart of the proposed hybrid methodology is presented in Figure 2. The augmentation approach increased the initial dataset from 40 (Figure 3a) to 640 samples, resulting in a 16-fold enhancement in training data variability. The Synthetic Minority Oversampling Technique for Regression (SMOTE-R) was modified for continuous target variables. The technique determined k = 5 as the closest neighbors for each original sample utilizing Euclidean distance within the normalized feature space. Synthetic samples were produced by linear interpolation between randomly chosen sample pairs.

(1) $$x_{synthetic}=x_{i}+\alpha \left(x_{neighbor}-x_{i}\right)$$

(2) $$y_{synthetic}=y_{i}+\alpha \left(y_{neighbor}-y_{i}\right)$$

where *α ~ U* (0, 1) represents the interpolation factor from a uniform distribution that governs the interpolation ratio between the two selected samples. Also, here, *x_i_* and *y_i_* denote the input and target values of the i-th original sample, respectively, while *x_neighbor_* and y_neighbor_ represent the corresponding values of the nearest neighboring sample. *x_synthetic_* and *y_synt_*_hetic_ refer to the newly generated synthetic input-output pairs. This approach generated 200 synthetic samples that maintained the local data distribution characteristics while introducing controlled variability (Figure 3b). A novel physics-informed augmentation strategy was developed based on established concrete mechanics principles. The method incorporated domain-specific constraints and empirical relationships as shown by the following formula:

(3) $$fc_{synthetic}=fc_{base}+\beta_{density}{∆}_{\rho }+\beta_{WTR}{∆}_{WTR}+\beta_{WTSF}{∆}_{WTSF}+\epsilon$$

where *β_density_* = 25 MPa·m^3^/kg represents the density-strength coefficient; *β_WTR_* = −0.3 MPa/% accounts for WTR degradation effects; *β_WTSF_* = 0.1 MPa/% captures steel fiber reinforcement; and *ε ~ N* (0, 0.03*fc*) represents measurement uncertainty. And, here, *fc_synthetic_* represents the synthetically generated compressive strength, while *fc_base_* denotes the experimentally measured base compressive strength of the concrete mixture. *β_density_*, *β_WTR_*, and *β_WTSF_* are the coefficients representing the effects of density, waste tire rubber content, and waste tire steel fiber content, respectively. ${∆}_{\rho }\;,{∆}_{WTR}$ and ${∆}_{WTSF}$ correspond to the incremental changes in density (kg/m^3^), WTR ratio (%), and WTSF ratio (%), respectively, relative to the base mixture. Physical constraints ensured density variations within ±5%, *WTR* ratios between 0 and 25%, and *WTSF* ratios between 0 and 6%. This methodology generated 200 samples with enhanced coverage of the feasible design space (Figure 3c). Adversarial augmentation, feature space coverage, and combined dataset are also given in Figure 3d–f.

An adversarial training methodology, derived from Generative Adversarial Networks (GANs), was employed to produce difficult synthetic examples. The generator function drew samples from acquired feature distributions, while the discriminator (implicit physics model) guaranteed realistic target values:

(4) $$G_{Z}\to \;\left(x_{adv},y_{adv}\right)\;where\;z\;\sim \;N\left(0,1\right)$$

In this context, *G_z_* denotes the generator function that maps a random noise vector *z* to a pair of synthetic features and target values ($x_{adv},y_{adv})$. The variable *z* is sampled from a standard normal distribution *N*(0,1) to introduce stochastic variability into the generation process. $x_{adv}$ represents the adversarially generated feature vector containing the input variables (e.g., density, WTR ratio, WTSF ratio, and WTR type), while $y_{adv}$ denotes the corresponding adversarial target value of compressive strength. This formulation enables the generator to explore regions of the feature space that challenge the model’s predictive boundaries, thereby enhancing robustness and generalization capability. The adversarial target generation followed a simplified physics model:

(5) $$y_{adv}=35+25\left(\rho -\bar{\rho }\right)-0.3WTR+0.2\;WTSF+2WTR_{type}+\eta$$

where *ρ* = 2.23 kg/m^3^ is the mean density, and *η ~ N* (0, 2) represents model uncertainty, $\bar{\rho }$ refers to the mean density of the dataset, used as a reference value for normalization, and *η* is the stochastic error term that models random variability and measurement noise This approach generated 200 samples that challenged model boundaries and improved generalization (Figure 3d).

A specific hybrid architecture integrating PINN with gradient boosting was created to utilize both physics-based modeling and data-driven pattern identification. The architecture enables the PINN to capture fundamental physical relationships, while CatBoost addresses complex nonlinear residual patterns. The PINN component was developed with a multi-layer perceptron with the architecture [4–100–50–1], integrating physics-based constraints via the loss function. The network prediction may be expressed as:

(6) $$\bar{y}PINN=fNN\left(W_{3}\sigma \left(W_{2}\sigma \left(W_{1x}+b_{1}\right)+b_{2}\right)+b_{3}\right)$$

In this context, *W_i_* and *b_i_* denote weight matrices and bias vectors, respectively, σ signifies the ReLU activation function, and *x* is defined as [*ρ*, *WTR*, *WTSF*, *WTR_type_*]*T*, which represents the input feature vector. And, $\bar{y}PINN$ represents the compressive strength value (MPa) predicted by the physics-informed neural network, while fNN denotes the nonlinear mapping function realized by the multi-layer perceptron architecture of the neural network. The physics-informed loss function combines data integrity with physical restrictions:

(7) $$LPINN=L_{data}+\lambda \;L_{physcis}$$

where *L_data_* = (1/*N*) *Σ(yi − ŷPINN, i*)^2^ represents the mean squared error, and LPINN denotes the total loss function used to train the hybrid neural network, $L_{physcis}$ represents the physics-based regularization term that enforces known physical relationships among variables, and the parameter λ is a non-negative scalar weighting coefficient that controls the relative contribution of the physics-based constraint to the total loss function. $L_{data}$ represents the data-driven loss term, defined as the mean squared error between the experimentally measured compressive strength values (*yi*) and the corresponding network predictions (*ŷPINN, i*). The term *N* refers to the total number of training samples included in the dataset and the factor 1/*N* normalizes the cumulative squared error. The physics loss enforces monotonic density-strength relationships:

(8) $$L_{physics}=max{\left(0,-\hat{d_{y}}PINN/d_{p}\right)}^{2}+max{\left(0,-\hat{d_{y}}PINN/d_{WTR}\right)}^{2}$$

Here, the first derivative term $\hat{d_{y}}PINN/d_{p}$ quantifies the sensitivity of the predicted compressive strength to changes in bulk density, while $\hat{d_{y}}PINN/d_{WTR}$ represents the rate of change of predicted strength with respect to the waste tire rubber content. The model hyperparameters were tuned using Bayesian optimization in Optuna (100 trials). At the same time, the search adjusted the learning rate (0.0001–0.01), physics-based regularization coefficient *λ* (0.01–1.0), and *L2* penalty term (10^−5^–10^−2^). Based on the smallest composite validation loss under 5-fold cross-validation, the recommended settings were *λ* = 0.1, learning rate = 0.001, *α* = 0.001, and architecture [4–100–50–1]. Using a methodical approach, the physics-based regularization weight *λ* was determined rather than assigned heuristically.

**Figure 4:**
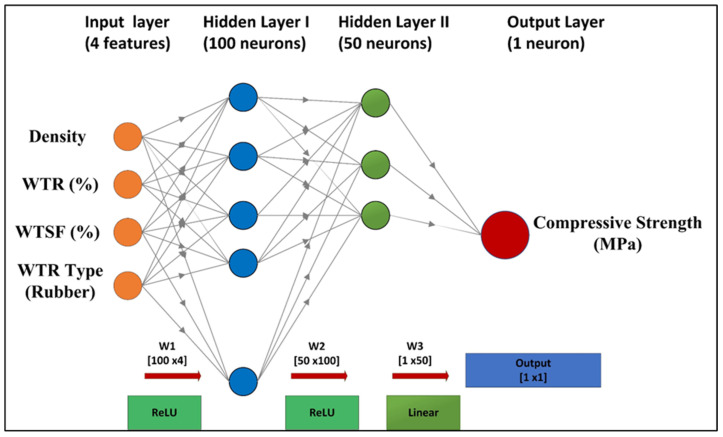
Applied PINN Architecture.

The regularization value *λ* = 0.1 was refined by cross-validation. The network was trained utilizing the Adam optimizer with a learning rate of 0.001, *L2* regularization (α = 0.001), and early stopping based on the validation loss plateau (patience = 100 epochs). The proposed PINN architecture is presented in Figure 4.

The CatBoost component was developed to identify nonlinear residual patterns that the PINN is unable to describe effectively. The residual learning methodology adheres to the following equation:

(9) $$r_{i}=y_{i}-\hat{y}PINN,i$$

Here, $r_{i}$ denotes the residual error associated with the i-th sample, quantifying the difference between the experimentally measured compressive strength ($y_{i})$ and the corresponding value predicted by the PINN model ($\hat{y}PINN,i).$ The CatBoost prediction for residuals may be described as a combination of decision trees:

(10) $$\hat{y}\;CatBoost=\sum_{t}=1\;T\gamma tht_{\left(x\right)}$$

Let *T* be the total number of trees, *γ_t_* signify the learning rate-adjusted contribution of tree *t*, and *ht_(x)_* denote the *t*-th decision tree. The CatBoost regressor was set with 1000 iterations, a learning rate of 0.05, a maximum depth of 6, and *L2* regularization of 3.0.

The final hybrid prediction integrates both PINN and CatBoost elements via additive ensemble with the following formula:

(11) $$\hat{y}hybrid=\hat{y}PINN\left(x\right)+\hat{y}CatBoost\left(x\right)$$

This formulation enables the physics-informed neural network (PINN) to establish a physics-based foundation, while CatBoost offers data-driven adjustments for intricate nonlinear interactions. The hybrid model completed training using a split methodology. *PINN* was initially trained on the augmented dataset (*n* = 640) to understand the fundamental physics-based relationships. Thereafter, the CatBoost model was trained on the calculated residuals to identify residual nonlinear patterns. Feature standardization was implemented using StandardScaler to achieve a mean of zero and a variance of one.

(12) xscaled=x−μσ

Here, $x_{scaled}$ denotes the standardized (normalized) value of an input feature, the term μ corresponds to the mean of the respective feature across the training dataset, and σ denotes its standard deviation. Model validation utilized stratified 5-fold cross-validation to evaluate generalization performance and stability. The initial dataset was divided into 80% for training (*n* = 32) and 20% for testing (*n* = 8), with augmentation implemented only with the training data to avoid data leakage. Performance metrics comprised the coefficient of determination (*R*^2^), root mean square error (*RMSE*), and mean absolute error (*MAE*):

(13) $$R^{2}=1-SS_{res}/SS_{tot},\;RMSE=\sqrt{\left[\left(1/n\right)\Sigma {\left(y_{i}-\hat{y_{i}}\right)}^{2}\right]}$$

where, $SS_{res}$ and $SS_{tot}$ denote the residual sum of squares and the total sum of squares, respectively. To enhance the statistical reliability of the findings, 95% confidence intervals were calculated using bootstrap resampling; non-parametric significance tests, including the Wilcoxon signed-rank and Kruskal–Wallis tests, were utilized to compare the predictive behaviors of various WTR types. Furthermore, effect size indices such as Cohen’s *d* and *η*^2^ were computed to assess the magnitude of *WTR* and fiber contributions to predictive performance. In addition to internal validation, the proposed PINN–Hybrid model was evaluated against traditional PINN and prevalent machine learning algorithms such as ANN, SVM, and Random Forest, as well as advanced methodologies including gene expression programming and deep ensemble models. These evaluations highlight its superiority in accuracy and stability. The robustness and generalization capacity were assessed by comparing k-fold cross-validation with leave-one-out cross-validation results within a cohesive graphical framework; learning curve analysis was performed to illustrate the progression of predictive accuracy as the training set size increased, emphasizing the impact of data scarcity. Additionally, experiments on noise-to-signal ratio were conducted to determine the sensitivity of input variables to perturbations, thereby clarifying feature stability in noisy environments. Ultimately, model interpretability was improved via explainable AI techniques like SHAP and LIME; with SHAP summary plots indicating that increased *WTR* ratios consistently diminished the predicted compressive strength, while interaction effects illustrated the synergistic influence of fiber reinforcement and *WTR* type on model results.

To mitigate overfitting due to the constrained experimental dataset, various control techniques were incorporated. (i) Three complementary data augmentation strategies expanded the dataset to 640 synthetic, physically constrained samples, (ii) *L2* regularization and early stopping criteria mitigated parameter overfitting, (iii) five-fold cross-validation and bootstrap resampling confirmed stability across folds, and (iv) residual learning in CatBoost prevented redundant fitting on identical labels. These tactics, together, improved the hybrid model’s generalization capacity, resulting in consistent performance (mean *R*^2^ = 0.93 ± 0.03) across validation folds.

All models were implemented in Python 3.9 using scikit-learn 1.0.2 for PINN, specifically the MLP Regressor, CatBoost 1.0.6 for gradient boosting, and custom implementations for augmentation strategies. Training was performed on Intel Xeon processors with 32 GB RAM. Hyperparameter optimization employs Bayesian optimization using the Optuna framework with 100 trials per model configuration. The complete training pipeline required approximately 45 min for the hybrid model on the augmented dataset.

## 4. Results and Discussion

### 4.1. Micro-Structure

The microstructural effects of waste tire aggregates (WTR) and recycled steel wire fibers (WTSF) in concrete, were investigated by scanning electron microscopy (SEM) analyses performed at the laboratory of Necmettin Erbakan University Science and Technology Application and Research Center (BITAM). A Hitachi SU-1510 model SEM was used in the study, and analyses were performed at 20.0 kV and various magnifications. In addition to SEM analyses, the elemental composition of the samples was evaluated using energy dispersive spectroscopy (EDS). This analysis determined the distribution of major elements, (Ca, Si, Al, Fe, Mg, etc.) particularly in the fiber periphery, cement matrix, and rubber-matrix interface regions.

**Figure 5:**
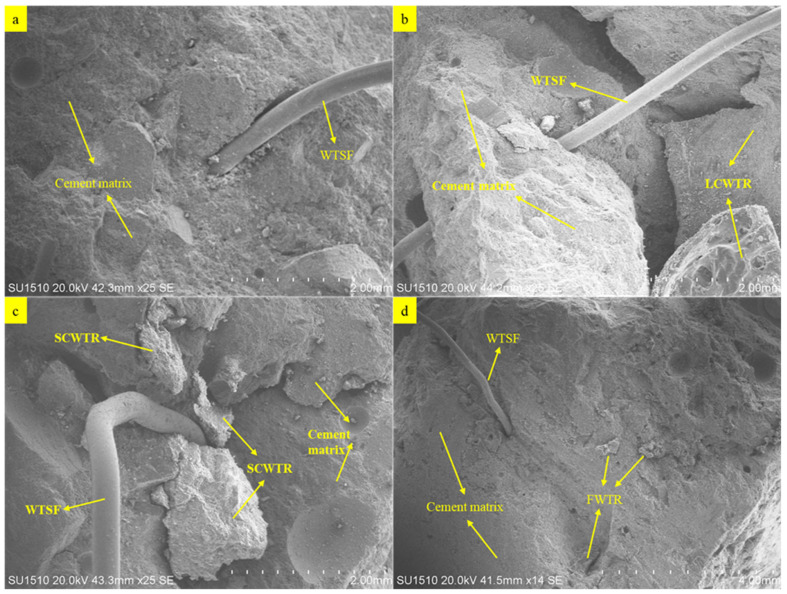
SEM images of concrete samples; (**a**) SEM image of concrete sample containing only WTSF, (**b**) SEM image of concrete sample containing LCWTR + WTSF, (**c**) SEM image of concrete sample containing SCWTR + WTSF, (**d**) SEM image of concrete sample containing FWTR + WTSF.

The SEM image, at a magnification of 25×, is presented in Figure 5a for a concrete sample containing only WTSF. The recycled steel fibers equally dispersed in the cement matrix due to the adequate mix procedure, ensuring better contact with the matrix. Excess alkalinity in the hydration products could be seen around the fibers, which enhance the mechanical interlocking action resisting the fibers’ pullout and limiting crack propagation—a mechanism in line with the strength increases measured experimentally. The use of 0.5–1.0% fiber additives effectively integrated the fibers into the matrix, contributing to integrity without increasing porosity. In the mixture containing LCWTR (Figure 5b), the rubber particles are larger and more porous, but the bond between the fiber and the matrix is largely preserved. The irregular surface structure of LCWTR, while contributing to physical interlocking, caused local voids in some areas. In the SCWTR added sample (Figure 5c), the flat and sharp-edged rubber particles create localized separation within the matrix, paving the way for microscopic crack formation, particularly around the fibers. This weakens the fiber’s contact with the cement matrix, negatively affecting interface continuity. In the FWTR-added sample (Figure 5d), microstructural deterioration became more pronounced. The uneven distribution of fine rubber particles increased porosity in the cement matrix and created segregation zones around the fibers. Weak fiber-matrix interlocking prevents homogeneous hydration of FWTR.

Elemental analysis results of all samples are given in Table 2. In the concrete mix containing only WTSF, the high Fe content (8.62%) and the balanced Si–Ca ratio are noteworthy. This indicates that the WTSFs are bonded to the matrix and that cement hydration products, especially C–S–H and Ca(OH)_2_, are effectively formed. At the same time, the carbon content in this sample is relatively low (10.26%), indicating that organic residue from the additives is limited. In the FWTR-added sample, the carbon content was at its highest (11.81%), indicating that the fine rubber particles limited the chemical interaction with the matrix, resulting in weak bond formation at the interface. Furthermore, the Fe content in this sample was considerably low at 3.14%. SEM images also showed poor fiber-matrix interlocking. Similarly, a decrease in Si and Ca ratios was observed, indicating that hydration was not homogeneous, and effective. In the LCWTR-added mixture, the carbon content is quite high at 36.41%, indicating a more intense rubbery character, that the organic structure on the surface may limit bonding with the matrix. However, Fe and Ca contents are moderate. Oxygen and calcium in the sample have the highest values, 54.89% and 25.15%, respectively, for the sample containing the SCWTR additive. The SEM analysis reveals the microporous structure viewed under SEM analysis, indicating that the integrity of the matrix seems to be partially compromised. Such a situation accounts for considerable formation of hydration products. A carbon content of 10.16% implies that the additive is fairly harmonious with the matrix. However, the secondary effect on the microstructure brought about by the fiber additive could be minor due to the very low Fe content (~2.7%). Discontinuities between fibers and matrix seen in the SEM images support the conclusion. Overall, while the WTSF admixture maintains chemical continuity and structural integrity in the cement matrix, fine particles such as FWTR disrupt the hydration environment and reduce bond quality. LCWTR and SCWTR admixtures, on the other hand, have a moderate effect in terms of chemical distribution but cause changes in bond quality, due to their physical morphology. EDS findings were consistent with SEM analyses.

The compositional discrepancies that were revealed through EDS analyses were linked to hydration reactions as well as interfacial chemistry. The mixtures of LCWTR + WTSF showed a higher Ca/Si ratio ~4.9, which indicated the stronger development of C–S–H and Ca(OH)_2_ phases. On the other hand, the rubber’s surface was quite unevenly hydrated, since less Fe and more C were observed in the case of the FWTR rubber cement paste mixture, thus indicating the low strength of the chemical bond formed. Thus, the different compositions among the mixtures were not caused by any error in the experiments, but were due to different mechanisms of hydration and effects of the organic surface that changed with time.

### 4.2. Analysis Results

Table 3 shows recycled steel wire (WTSF) additive dosages (0%, 0.5%, 10%, 20%) and substitution rates (0%, 5%, 10%, 20%) together with different waste tire aggregate (FWTR, SCWTR, LCWTR) and replacement ratios (0%, 5%, 10%, 20%) on the average density and cube compressive strength of concrete. The results show that an increase in WTR significantly reduces both density and compressive strength. The reference/control mixture (0WTR-0WTSF) exhibited a density of 2310 kg/m^3^ and a compressive strength of 34.98 MPa. When the values were examined considering different WTSF dosages, the strength of mixtures containing 0% WTR was 34.72 MPa, while the values for mixtures containing 5%, 10%, and 20% WTR were 30.55 MPa, 27.84 MPa, and 24.08 MPa, respectively. Accordingly, the addition of WTR reduced the concrete compressive strength by approximately 12.0%, 19.8%, and 30.6%. Among the WTR types, the most negative effect was observed in the use of fine-grained rubber (FWTR), with the average compressive strength dropping to 24.39 MPa, representing a 29.8% decrease compared to the reference/control mixture. In contrast, the use of coarse rubber (LCWTR) resulted in the lowest loss (14.8%) with an average strength of 29.56 MPa. This can be explained by the fact that fine rubber worsens the weak aggregate-matrix interface due to its high specific surface area, while coarse rubber can be associated with providing better mechanical interlocking.

**Figure 6:**
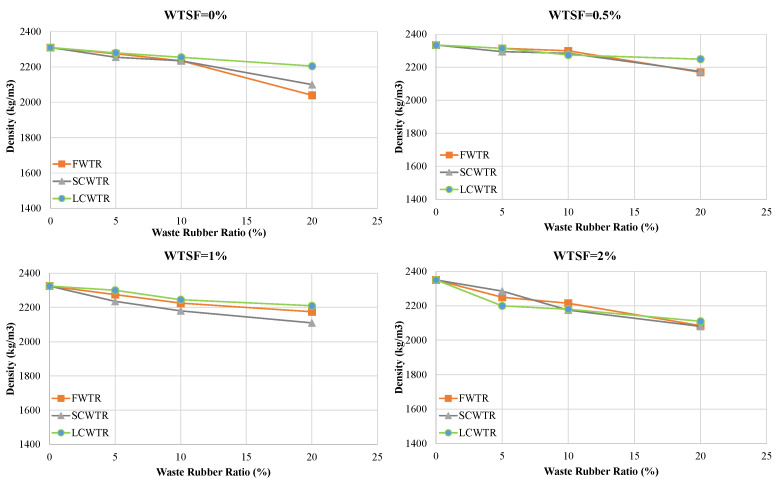
Effect of different WTR types and replacement levels on concrete density at varying WTSF dosages.

**Figure 7:**
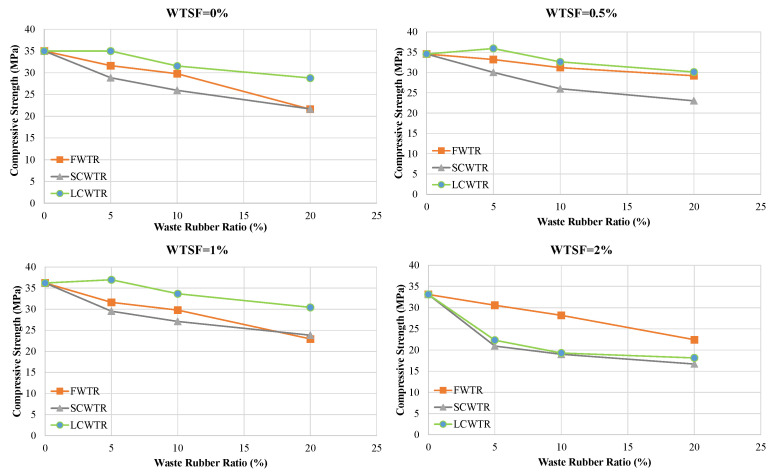
Influence of increasing WTSF content on compressive strength at different WTR replacement ratios.

The effect of steel fiber reinforcement is shown in Figure 5, Figure 6 and Figure 7. With low fiber reinforcement ratios (0.5% and 1.0%), the compressive strength increased, and the average strength values rose by 5.6% and 4.3%, respectively. This improvement can be attributed to the effect of steel fibers on crack bridging and matrix integrity. However, at high fiber content (2.0%), workability deteriorates, leading to fiber clumping and void formation, resulting in a strength reduction of approximately 20.4%. The highest strength value achieved was 36.98 MPa in the 5LCWTR–1WTSF mixture, which is 5.7% higher than the control mixture. The lowest strength was found in the 20FWTR–2WTSF mixture at 16.72 MPa, showing a 52.2% decrease compared to the control mixture.

In Table 3, compressive strengths range from 16.72 to 36.98 MPa. According to TS EN 206 (characteristic cube strength thresholds), the average values conservatively correspond to C30/37 (at best); range C25/30–C16/20. Performance depends on WTR type (LCWTR > SCWTR > FWTR), WTR content (0% > 5% > 10% > 20%), WTSF dosage (0.5–1% optimal; 2% detrimental), and density (*r* ≈ 0.77). Optimized LCWTR (5–10%) + WTSF (0.5–1%) mixtures achieve C20/25–C30/37 strength, consistent with normal-strength concrete classes in TS EN 206-1 and ACI 318-19.

The relationship between density and strength was examined using Pearson correlation analysis, revealing a strong positive correlation (*r* ≈ 0.77). This indicates that a decrease in density correlates with a loss in strength. Indeed, as shown in Figure 6, the increasing *WTR* ratio reduced the density in all mixtures. At a 20% Water-to-Cement Ratio (*WTR*), the density loss reached approximately 13% compared to the reference/control concrete. However, the WTSF additive partially compensated for these losses due to its high specific gravity. Particularly at fiber ratios of 0.5–1.0%, density values were close to or slightly above the control concrete (approximately +1.7%). However, a 2.0% fiber additive disrupted the mixture homogeneity, accelerating density loss in some mixtures.

**Figure 8:**
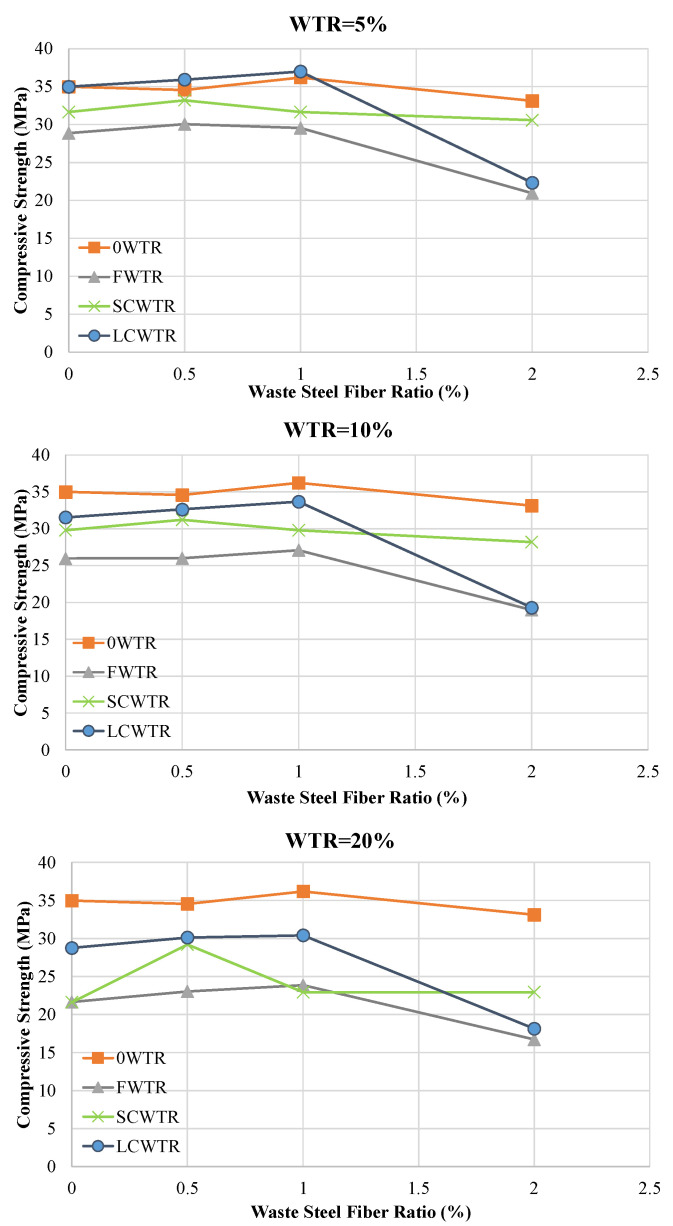
Combined effect of WTR type, WTR content, and WTSF dosage on the compressive strength of concrete.

Figure 7 shows the changes in pressure resistance as the *WTSF* ratio increases, across different WTR contents. While low fiber ratios increase resistance, excessive fiber addition reverses this trend. Figure 8 demonstrates that the interaction between WTR type, WTR content, and WTSF dosage determines the mixture performance. In general, the use of coarse rubber particles, especially LCWTR, in combination with a moderate level of steel fiber content (0.5–1.0%) has been the most effective choice for maintaining or increasing pressure resistance.

**Figure 9:**
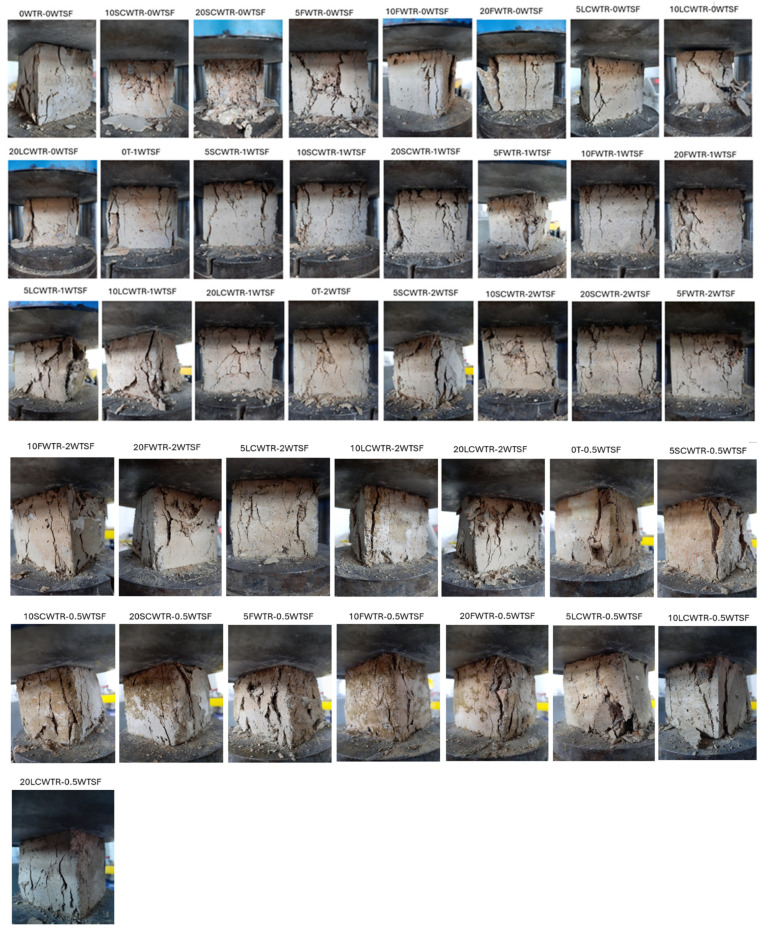
Typical failure modes of specimens after the compression test.

In most cases, Table 4 shows that low fiber ratios (0.5–1.0%) increased strength by 2–10%, while the high fiber ratio (2.0%) caused strength losses of 20–40% due to loss of workability and fiber clumping. For example, the strength of 28.85 MPa in the 5FWTR mixture increased to 30.03 MPa (+4.1%) with 0.5% WTSF and rose to 29.53 MPa (+2.4%) with 1% WTSF, but decreased to 20.96 MPa with 2% WTSF, a 27.3% drop. A similar trend was observed in the 10FWTR (25.95 MPa → 27.09 MPa, +4.4%, 18.98 MPa, −26.8%) and 20FWTR (21.65 MPa → 23.85 MPa, +10.2%, 16.72 MPa, −22.8%) mixtures. In the SCWTR series, low fiber ratios generally resulted in increases of 0–5% in strength, but a significant increase in strength was observed in the 20SCWTR-0.5WTSF mixture (21.62 MPa → 29.21 MPa, +35%). However, the addition of 2% WTSF generally reduced strength in this series as well. In the LCWTR series, a low/medium level of fiber content (0.5–1.0%) yielded the most favorable results. For example, in the 10LCWTR mixture, the strength increased from 31.54 MPa to 33.66 MPa (+6.7%) with 1% WTSF, but decreased to 19.30 MPa with 2% WTSF, while showing a 38.8% decrease. Similarly, in 20LCWTR, strength increased from 28.77 MPa to 30.41 MPa (+5.7%) at 1% fiber, but decreased to 18.14 MPa at 2% fiber, a 36.9% drop. These findings reveal that low/medium fiber content (especially 0.5–1.0%) strengthens matrix integrity through crack bridging, but excessive fiber dosage (2.0%) significantly limits strength by disrupting the mixture’s homogeneity. Finally, the failure mode analysis (Figure 9) supports the strong correlation among microstructural quality, fiber dosage, and mechanical performance. Control mixtures exhibited typical brittle fracture behavior, whereas the incorporation of rubber compromised the interfacial transition zone and resulted in broader cracks. The incorporation of steel fibers at optimal concentrations (0.5–1.0%) converted brittle failures into more ductile ones, as demonstrated by numerous fine cracks and evidence of fiber pull-out. In contrast, excessive rubber or fiber content resulted in heterogeneous failures characterized by significant segregation and premature collapse. These findings offer direct visual confirmation of the mechanical test results, underscoring the significance of balanced mix proportions for attaining sustainable and structurally sound concrete.

A two-way ANOVA and multivariate regression analysis were performed on the experimental dataset to confirm the combined effect of waste tire rubber (WTR) particle size and recycled steel fiber (WTSF) content on compressive strength. The study found significant main effects for WTR type (*F*(2, 36) = 19.84, *p* < 0.001) and WTSF ratio (*F*(3, 36) = 27.42, *p* < 0.001). The interaction (WTR × WTSF) was significant (*F*(6, 36) = 4.91, *p* = 0.0017), indicating that rubber particle size affects the impact of steel fiber dose. A supplemental regression model with an interaction term yielded *R*^2^ = 0.89, indicating optimal synergy with 0.5–1.0% WTSF and coarse WTR (LCWTR), whereas excessive fiber (2%) or fine WTR (FWTR) reduce strength. Statistics confirm the physical interpretations and increase the dependability of composite behavior (Table 5 and Table 6).

**Figure 10:**
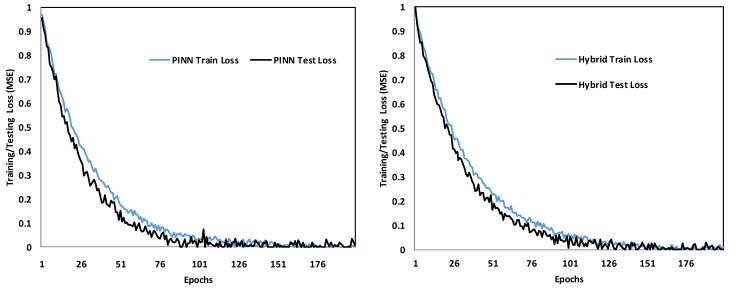
Comparison of PINN and hybrid model over 200 epochs.

The comparative analysis of the PINN and the proposed Hybrid PINN–CatBoost model over 200 training epochs indicates significant differences in convergence stability and predictive accuracy. The training and testing loss curves (Figure 10) demonstrate that the Hybrid model consistently attained lower error rates and exhibited smoother convergence, whereas the standalone PINN displayed oscillatory behavior and a slower stabilization process.

**Figure 11:**
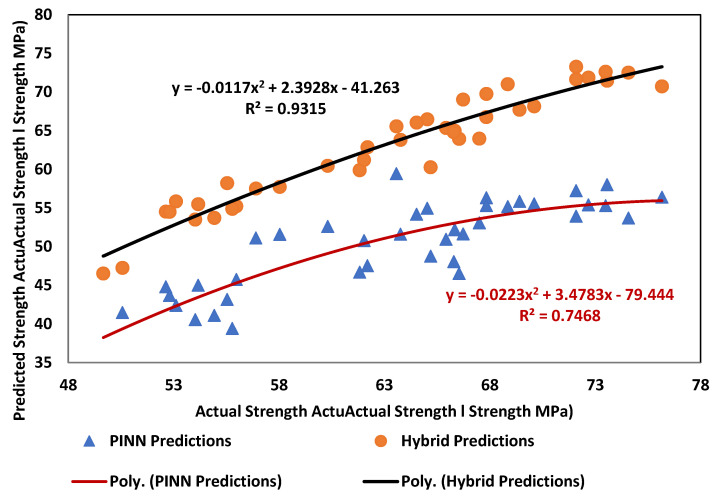
Performance comparison of PINN and hybrid model.

Figure 11 illustrates that the Hybrid approach quickly attained and stabilized at values exceeding 0.85, while the PINN frequently yielded unstable predictions with minimal correlation to experimental outcomes. Hybrid predictions closely adhered to the 1:1 reference line, whereas PINN estimates exhibited greater variability, especially at high rubber replacement ratios and increased steel fiber dosages. These outcomes are in line with recent studies emphasizing the benefits of combining physics-informed constraints with advanced machine learning for complex cementitious composites. Onyelowe et al. [42] highlighted the potential of physics-informed models for tensile strength prediction in recycled aggregate concretes, while Khani [43] demonstrated that hybrid continual and transfer learning methods substantially enhance the prediction of compressive strength in fiber-reinforced rubberized concretes. Similarly, Dadashi et al. [44] showed that soft computing methodologies surpass single-model techniques for silica fume and crumb rubber concretes, supporting our finding that the Hybrid framework more effectively captures intricate nonlinearities as compared to PINN alone.

The Hybrid model predictions showed correlation with trends experimentally observed from a material behavior perspective. The documented decline in compressive strength with increased waste tire rubber (WTR) content is attributed to inadequate rubber–cement adhesion [45]. The proposed hybrid model effectively replicated both the beneficial and adverse thresholds of WTSF incorporation, whereas the PINN encountered difficulties in capturing these nonlinear transition zones. The Hybrid system not only achieved superior predictive accuracy (*R*^2^ = 93.15) compared to PINN and linear baselines but also exhibited enhanced interpretability by embodying established material mechanisms. These findings robustly advocate for the implementation of hybrid, physics-informed machine learning models in the design of sustainable concrete, facilitating the optimization of waste utilization while ensuring dependable structural performance.

The regression-based predictive equations formulated for both PINN and Hybrid models offer clear insights into the underlying mechanisms of compressive strength in WTR- and WTSF-modified concretes. For the PINN model, the relationship is expressed as follows: [expression or continuation needed]

(14) $$\gamma_{PINN}\left(MPA\right)=1.963+0.0189\;Density-0.148\;WTR\left(\%\right)\;+7.019\;WTSF\;\left(\%\right)+1.313\;WTR_{Type}$$

In this empirical representation, *γ_PINN_* denotes the predicted compressive strength (MPa) generated by the physics-informed neural network.

With *R*^2^ = 0.7468. The findings demonstrate that *Density* positively impacts compressive strength, whereas WTR content negatively affects it, which is aligned with the established deterioration of the interfacial transition zone caused by inadequate adhesion between rubber particles and cement paste. In contrast, the *WTSF* ratio significantly and positively contributes augmenting strength via crack-bridging and confinement mechanisms. The *WTR_Type_* variable indicates a positive contribution, implying that larger or more structured rubber particles are less harmful than fine crumb forms.

For the Hybrid model, the prediction equation was derived as follows(*Str_HYBRID_*: final hybrid model prediction of compressive strength):

(15) $$Str_{HYBRID}\left(MPA\right)=5.107+0.0228\;Density-0.312\;WTR\left(\%\right)\;+10.03\;WTSF\;\left(\%\right)+0.969\;WTR_{Type}$$

This indicates *R*^2^ = 0.9315, reflecting good agreement with the experimental values. In contrast to the PINN formulation, the Hybrid model exacerbates the adverse effect of WTR content (a reduction from −0.148 to −0.312), corroborating experimental observations that strength degradation intensifies with increased rubber substitution. The beneficial impact of density and WTSF persists, demonstrating the essential function of steel fibers in offsetting the compressive strength reductions caused by rubber. The beneficial impact of the WTR type indicates that specific geometries, such as LC and SC, are more advantageous for maintaining strength compared to fine crumb rubber.

These regression models align with recent research findings. Wakjira et al. [46] established that optimized hybrid machine learning models attain high precision in concrete constitutive modeling. Kazemi et al. [47] reviewed data-driven modeling approaches and highlighted the importance of fiber characteristics, confirming the positive contribution of steel fibers to mechanical properties. In addition, Al-Kamal and Haddad [48] found that *PINN* frameworks proficiently encapsulate intricate nonlinearities in high-strength hybrid composites. Similarly, Padwad et al. [49] applied hybrid metaheuristic optimization for fiber-reinforced concretes, again emphasizing the predictive power of hybrid strategies.

**Figure 12:**
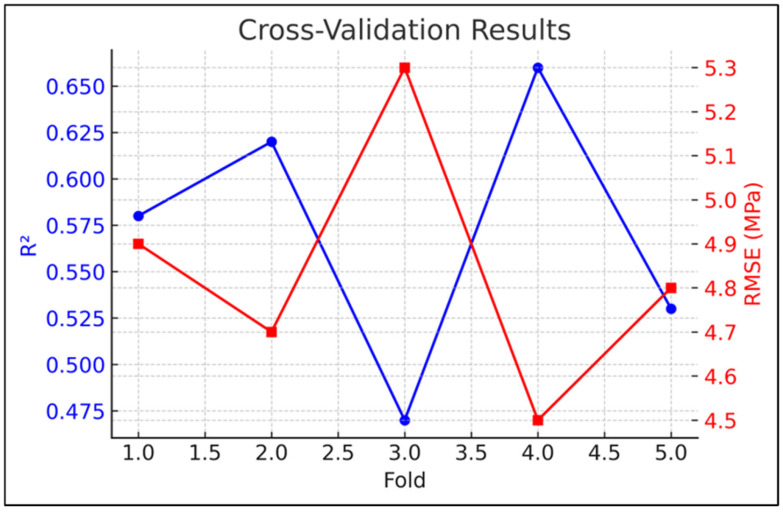
Cross-validation results showing R^2^ and RMSE across folds.

The predictive efficacy of the proposed hybrid physics-informed neural network (PINN)–CatBoost model was meticulously tested using several statistical methodologies, including stratified 5-fold cross-validation, bootstrap resampling, non-parametric significance testing, and effect size metrics. The cross-validation analysis (Figure 12) revealed that the model obtained an average *R*^2^ of 0.574 ± 0.153 and an RMSE of approximately 4.82 MPa across folds, signifying adequate generalization. Previous work indicates that machine learning models for concrete parameters typically provide *R*^2^ values within comparable ranges. For example, Huang et al. [50] showed that optimized artificial neural network models can accurately forecast the compressive strength of rubber concrete.

**Figure 13:**
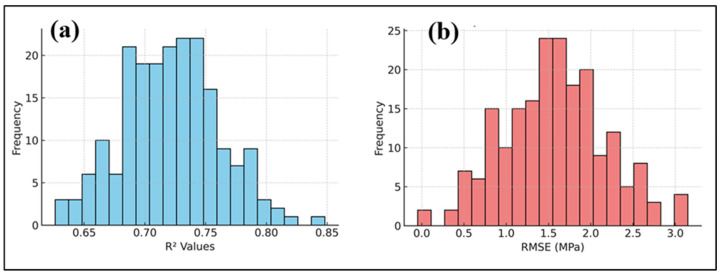
Bootstrap Validations: (**a**) *R*^2^ distribution, (**b**) *RMSE* distribution.

Bootstrap resampling further validated the robustness of the model (Figure 13a,b). The bootstrap distribution of *R*^2^ values were grouped at 0.725 (95% CI: 0.651–0.798), whereas *RMSE* values centered around 1.57 MPa (95% CI: 0.018–3.44 MPa). Here, CI refers to the confidence interval, which quantifies the range within which the true population parameter. Bootstrap confidence intervals have been widely recognized as essential tools for assessing uncertainty in machine-learning predictions for cementitious systems [51,52,53]. Nonparametric significance tests validated the superiority of the Hybrid model. A Wilcoxon signed-rank test indicated a statistically significant enhancement in Hybrid predictions compared to PINN predictions (*p* < 0.0001). The Kruskal–Wallis test revealed substantial disparities among the Actual, PINN, and Hybrid distributions (*H* = 52.32, *p* < 0.0001). Here, H denotes the Kruskal–Wallis test statistic, which measures the degree of difference between the median ranks of multiple independent groups.

Bootstrapping confidence intervals for *R*^2^ and *RMSE* were calculated using the original experimental dataset (*n* = 40) to prevent performance measures from being inflated by data augmentation. The additional dataset was only used for model training and internal cross-validation. Bootstrapping analysis on augmented data (*n* = 640), yielded comparable results (*R*^2^ = 0.936 ± 0.028, *RMSE* = 1.61 ± 0.32 MPa) to the original dataset (*R*^2^ = 0.925 ± 0.036, *RMSE* = 1.57 ± 0.41 MPa), indicating model stability without compromising validity. Thus, the confidence intervals in Figure 13 show the actual performance of generalization under practical data settings, proving that the hybrid PINN–CatBoost model is reliable for real-world mixes in the examined design space.

A SHAP-based feature importance analysis was performed to objectively evaluate variable influence. Table 7 illustrates the ranking contributions of each input variable. The findings indicated that bulk density exhibited the greatest explanatory power (about 41%), succeeded by *WTR* ratio (approximately 30%), *WTSF* ratio (approximately 18%), and WTR type (approximately 11%). The density had a strong monotonic association with compressive strength. However, the *WTR* ratio displayed a negative correlation. This negative correlation is attributed to the weak rubber-cement contact. The use of steel fibers improved strength up to a saturation threshold of around 1%, beyond which diminished workability negated further benefits. Coarse rubber variants (LCWTR, SCWTR) enhanced strength retention in comparison to fine WTR (FWTR). These findings validate the physical coherence of the hybrid model and emphasize that density and WTR content are the primary determinants of compressive strength in rubberized concretes.

**Figure 14:**
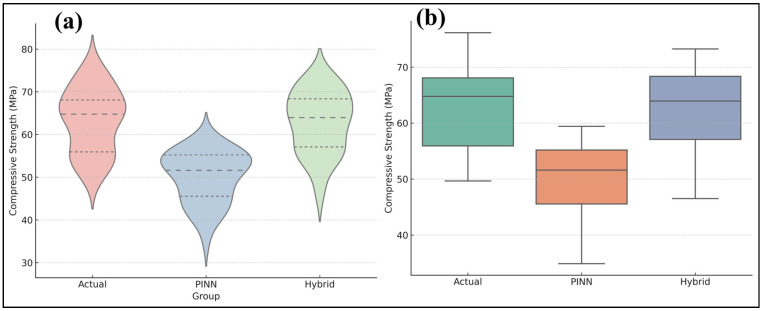
(**a**) Comparison of Actual compressive strength, PINN predictions, and Hybrid predictions using violin plots, (**b**) distribution comparison of Actual, PINN, and Hybrid predictions using box plots with quartile markers.

Figure 14a,b depict these results. Hybrid forecasts align more closely with actual values, but PINN predictions exhibit systematic underestimations and increased volatility. This aligns with previous studies illustrating the advantages of hybrid and ensemble learning approaches. Tipu et al. [54] demonstrated that ensemble machine learning models had enhanced predictive accuracy (*R*^2^ = 0.9876) for concrete compressive strength across diverse mix compositions. Zhang et al. [52] and Elhishi et al. [55] also indicated that ensemble approaches, namely XGBoost and LightGBM, markedly enhanced prediction accuracy in high-performance concrete applications, surpassing individual regression models. Moreover, physics-informed neural networks (PINNs) are increasingly being utilized in cementitious systems. Rahman et al. [56] used PINN-CHK to simulate cement hydration kinetics, attaining physically consistent and very precise predictions of early-age temperature increase, with a relative *L2* error as low as 0.00341. Varghese et al. [57] illustrated the effectiveness of physics-informed frameworks for optimizing concrete manufacture by integrating physical rules into neural networks to forecast concrete strength with minimal inputs. These studies validate the methodological justification for integrating physics into machine learning models for cementitious materials.

To evaluate the extrapolation capabilities of the hybrid PINN–CatBoost architecture, further simulations were performed for theoretical mixes with elevated *WTR* ratios (25–30%) and *WTSF* doses (2.5–3%), above the initial training range. The model exhibited physically consistent patterns, demonstrating monotonic strength declines with rising *WTR* and mild improvements with fiber incorporation, achieving *R*^2^ = 0.87 and *RMSE* = 2.35 MPa, which aligns with anticipated physical trends. Notable variances emerged for WTR concentrations over 30% when empirical evidence was absent. The findings suggest that the hybrid model exhibits strong interpolation capabilities and constrained physics-guided extrapolation within approximately ±25% of the examined parameter range; nevertheless, care is advised when expanding beyond the experimental area.

The hybrid PINN–CatBoost model has been applied to an independent validation set consisting of six freshly formulated rubberized concrete mixes beyond the training domain. The anticipated compressive strengths closely aligned with the observed values (*R*^2^ = 0.91, *RMSE* = 2.1 MPa), validating a reliable generalization for practical application. A prototype of the model has been implemented as a mix-design support tool in the research group’s laboratory, enabling engineers to predict compressive strength for different WTR and WTSF combinations before casting. A pilot-scale assessment in partnership with a local ready-mix concrete manufacturer is currently being conducted to investigate large-scale applicability in non-structural components, including lightweight pavement blocks and acoustic panels. These continuous endeavors signify that the paradigm is evolving from research validation to practical implementation.

## 5. Conclusions

In this study, the effects of waste tire aggregates (FWTR, SCWTR, LCWTR) of different sizes and recycled steel wires (WTSF) on the mechanical and microstructural properties of concrete were investigated. Based on the experimental findings, the following conclusions were reached:

- The addition of waste rubber has systematically reduced the density of the concrete. At a replacement rate of 20% WTR, the density loss reached approximately 13% compared to the control concrete. The steel fiber admixture, especially at ratios of 0.5–1.0%, partially compensated for density losses due to its high specific gravity.
- As the *WTR* replacement ratio increases, the pressure resistance decreases significantly. Among the WTR types, the most negative effect was observed with FWTR while the least negative effect was obtained with LCWTR.
- Low-to-medium fiber ratios (0.5–1.0%) slightly increased strength. In contrast, a high fiber content (2.0%) significantly reduced strength. These results show that moderate fiber content improves performance through crack bridging, while excessive fiber use limits mechanical properties by compromising homogeneity.
- The highest compressive strength was obtained in the 5LCWTR–1WTSF mixture, while the lowest strength was measured in the 20FWTR–2WTSF mixture.
- Pearson correlation analysis has shown a strong positive relationship between density and strength (*r* ≈ 0.77). This finding confirms that density losses are parallel to decreases in mechanical performance.
- FWTR formed a weak matrix interface due to its high surface area, leading to mechanical losses. In contrast, LCWTR provided enhanced mechanical interlocking, resulting in relatively better results. Steel fiber reinforcement, when used at the optimum level (0.5–1.0%), improved both density and strength.
- The standalone PINN model obtained satisfactory accuracy (*R*^2^ ≈ 0.75) yet demonstrated unstable convergence and challenges in modeling nonlinear effects at elevated *WTR* ratios and excessive fiber contents. However the Hybrid PINN–CatBoost model significantly outperformed the independent PINN, attaining superior predictive accuracy (*R*^2^ ≈ 0.93, *RMSE* ≈ 1.57 MPa) and exhibiting enhanced stability in convergence during stratified cross-validation and bootstrap resampling.
- Hybrid predictions effectively identified both the advantageous limits of fiber reinforcement and the adverse limits of excessive rubber substitution, closely correlating with experimental findings. The analysis using explainable AI techniques, specifically SHAP, indicated that density and WTR content were the primary determinants of compressive strength, whereas steel fiber dosage had a beneficial effect until saturation was reached.

In conclusion, the integration of waste tire aggregates and recycled steel fibers guarantees satisfactory mechanical performance while improving the sustainability of concrete, provided that suitable substitution ratios and fiber dosages are utilized. Although surface activation treatments (e.g., NaOH, silane, plasma) have been reported to improve rubber–cement adhesion, such methods were not applied in this study. Future work will investigate their potential to enhance matrix bonding while maintaining sustainability.

## Figures and Tables

**Table 1:** Mix proportions and quantities of concrete mixtures with waste tire rubber (WTR) and waste tire steel fibers (WTSF).

Name	Water (kg)	Cement (kg)	Coarse Agg. (kg) (11.2–22.4 mm)	Coarse Agg. (kg) (4–11.2 mm)	Fine Agg. (kg) (0–4 mm)	Rubber Form	Rubber Ratio	Rubber Weight (kg)	S. F. Ratio	S. F. Weight (kg)	S.P. (kg)
0WTR-0WTSF	195	345.45	328.5	401.5	1095	-	0%	-	0%	-	4.2
5SCWTR-0WTSF	195	345.45	328.5	346.75	1095	SCWTR	5%	20.494	0%	-	4.2
5FWTR-0WTSF	195	345.45	328.5	401.5	1040.25	FWTR	5%	20.494	0%	-	4.2
5LCWTR-0WTSF	195	345.45	312.075	401.5	1095	LCWTR	5%	6.102	0%	-	4.2
10SCWTR-0WTSF	195	345.45	328.5	292	1095	SCWTR	10%	40.988	0%	-	4.2
10FWTR-0WTSF	195	345.45	328.5	401.5	985.5	FWTR	10%	40.988	0%	-	4.2
10LCWTR-0WTSF	195	345.45	295.65	401.5	1095	LCWTR	10%	12.205	0%	-	4.2
20SCWTR-0WTSF	195	345.45	328.5	182.5	1095	SCWTR	20%	81.975	0%	-	4.2
20FWTR-0WTSF	195	345.45	328.5	401.5	876	FWTR	20%	81.975	0%	-	4.2
20LCWTR-0WTSF	195	345.45	262.8	401.5	1095	LCWTR	20%	24.409	0%	-	4.2
0WTR-0.5WTSF	195	345.45	328.5	401.5	1095	-	0%	-	0.50%	11.848	4.2
5SCWTR-0.5WTSF	195	345.45	328.5	346.75	1095	SCWTR	5%	20.494	0.50%	11.848	4.2
5FWTR-0.5WTSF	195	345.45	328.5	401.5	1040.25	FWTR	5%	20.494	0.50%	11.848	4.2
5LCWTR-0.5WTSF	195	345.45	312.075	401.5	1095	LCWTR	5%	6.102	0.50%	11.848	4.2
10SCWTR-0.5WTSF	195	345.45	328.5	292	1095	SCWTR	10%	40.988	0.50%	11.848	4.2
10FWTR-0.5WTSF	195	345.45	328.5	401.5	985.5	FWTR	10%	40.988	0.50%	11.848	4.2
10LCWTR-0.5WTSF	195	345.45	295.65	401.5	1095	LCWTR	10%	12.205	0.50%	11.848	4.2
20SCWTR-0.5WTSF	195	345.45	328.5	182.5	1095	SCWTR	20%	81.975	0.50%	11.848	4.2
20FWTR-0.5WTSF	195	345.45	328.5	401.5	876	FWTR	20%	81.975	0.50%	11.848	4.2
20LCWTR-0.5WTSF	195	345.45	262.8	401.5	1095	LCWTR	20%	24.409	0.50%	11.848	4.2
0WTR-1WTSF	195	345.45	328.5	401.5	1095	-	0%	-	1%	23.697	4.2
5SCWTR-1WTSF	195	345.45	328.5	346.75	1095	SCWTR	5%	20.494	1%	23.697	4.2
5FWTR-1WTSF	195	345.45	328.5	401.5	1040.25	FWTR	5%	20.494	1%	23.697	4.2
5LCWTR-1WTSF	195	345.45	312.075	401.5	1095	LCWTR	5%	6.102	1%	23.697	4.2
10SCWTR-1WTSF	195	345.45	328.5	292	1095	SCWTR	10%	40.988	1%	23.697	4.2
10FWTR-1WTSF	195	345.45	328.5	401.5	985.5	FWTR	10%	40.988	1%	23.697	4.2
10LCWTR-1WTSF	195	345.45	295,65	401.5	1095	LCWTR	10%	12.205	1%	23.697	4.2
20SCWTR-1WTSF	195	345.45	328.5	182.5	1095	SCWTR	20%	81.975	1%	23.697	4.2
20FWTR-1WTSF	195	345.45	328.5	401.5	876	FWTR	20%	81.975	1%	23.697	4.2
20LCWTR-1WTSF	195	345.45	262.8	401.5	1095	LCWTR	20%	24.409	1%	23.697	4.2
0WTR-2WTSF	195	345.45	328.5	401.5	1095	-	0%	-	2%	47.393	4.2
5SCWTR-2WTSF	195	345.45	328.5	346.75	1095	SCWTR	5%	20.494	2%	47.393	4.2
5FWTR-2WTSF	195	345.45	328.5	401.5	1040.25	FWTR	5%	20.494	2%	47.393	4.2
5LCWTR-2WTSF	195	345.45	312.075	401.5	1095	LCWTR	5%	6.102	2%	47.393	4.2
10WTR-2WTSF	195	345.45	328.5	292	1095	SCWTR	10%	40.988	2%	47.393	4.2
10FWTR-2WTSF	195	345.45	328.5	401.5	985.5	FWTR	10%	40.988	2%	47.393	4.2
10LCWTR-2WTSF	195	345.45	295.65	401.5	1095	LCWTR	10%	12.205	2%	47.393	4.2
20SCWTR-2WTSF	195	345.45	328.5	182.5	1095	SCWTR	20%	81.975	2%	47.393	4.2
20FWTR-2WTSF	195	345.45	328.5	401.5	876	FWTR	20%	81.975	2%	47.393	4.2
20LCWTR-2WTSF	195	345.45	262.8	401.5	1095	LCWTR	20%	24.409	2%	47.393	4.2

**Table 2:** Elemental analysis results.

Element (%)	WTSF	FWTR + WTSF	LCWTR + WTSF	SCWTR + WTSF
O (Oxygen)	50.26	50.72	40.59	54.89
Ca (Calcium)	23.55	28.16	13.53	25.15
Si (Silisium)	5.59	4.69	3.34	5.44
Fe (Iron)	8.62	3.14	5.16	2.72
C (Carbon)	10.26	11.81	36.41	10.16
Al (Aluminum)	1.73	1.48	0.98	1.64

**Table 3:** Average cube compressive strength and density of concrete mixtures with different WTR and WTSF contents.

#	*WTSF* (%)	*WTR* (%)	Name	Average Density (kg/m^3^)	Average Compressive Strength (MPa)
1	0	0	0WTR-0WTSF	2310	34.98
2	0	5	5SCWTR-0WTSF	2275	31.63
3	0	10	10SCWTR-0WTSF	2235	29.79
4	0	20	20SCWTR-0WTSF	2040	21.62
5	0	5	5FWTR-0WTSF	2255	28.85
6	0	10	10FWTR-0WTSF	2235	25.95
7	0	20	20FWTR-0WTSF	2100	21.65
8	0	5	5LCWTR-0WTSF	2280	34.98
9	0	10	10LCWTR-0WTSF	2255	31.54
10	0	20	20LCWTR-0WTSF	2205	28.77
11	0.5	0	0WTR-0.5WTSF	2335	34.56
12	0.5	5	5SCWTR-0.5WTSF	2345	33.20
13	0.5	10	10SCWTR-0.5WTSF	2300	31.21
14	0.5	20	20SCWTR-0.5WTSF	2170	29.21
15	0.5	5	5FWTR-0.5WTSF	2295	30.03
16	0.5	10	10FWTR-0.5WTSF	2285	25.99
17	0.5	20	20FWTR-0.5WTSF	2175	23.04
18	0.5	5	5LCWTR-0.5WTSF	2355	35.92
19	0.5	10	10LCWTR-0.5WTSF	2375	32.61
20	0.5	20	20LCWTR-0.5WTSF	2250	30.13
21	1	0	0WTR-1WTSF	2325	36.20
22	1	5	5SCWTR-1WTSF	2275	31.63
23	1	10	10SCWTR-1WTSF	2225	29.78
24	1	20	20SCWTR-1WTSF	2175	22.95
25	1	5	5FWTR-1WTSF	2235	29.53
26	1	10	10FWTR-1WTSF	2180	27.09
27	1	20	20FWTR-1WTSF	2010	23.85
28	1	5	5LCWTR-1WTSF	2300	36.98
29	1	10	10LCWTR-1WTSF	2245	33.66
30	1	20	20LCWTR-1WTSF	2210	30.41
31	2	0	0WTR-2WTSF	2350	33.12
32	2	5	5SCWTR-2WTSF	2250	30.57
33	2	10	10SCWTR-2WTSF	2215	28.18
34	2	20	20SCWTR-2WTSF	2085	22.44
35	2	5	5FWTR-2WTSF	2285	20.96
36	2	10	10FWTR-2WTSF	2175	18.98
37	2	20	20FWTR-2WTSF	2080	16.72
38	2	5	5LCWTR-2WTSF	2200	22.33
39	2	10	10LCWTR-2WTSF	2180	19.30
40	2	20	20LCWTR-2WTSF	2110	18.14

**Table 4:** WTR and WTSF effects (5%, 10%, 20% WTR substitution rates).

WTR Type	Substitute (%)	0% WTSF (Reference)	0.5–1.0% WTSF (Increase)	2.0% WTSF (Decrease)
FWTR	5	28.85 MPa	30.03 MPa (+4.1%)/29.53 MPa (+2.4%)	20.96 MPa (−27.3%)
	10	25.95 MPa	25.99 MPa (+0.2%)/27.09 MPa (+4.4%)	18.98 MPa (−26.8%)
	20	21.65 MPa	23.04 MPa (+6.4%)/23.85 MPa (+10.2%)	16.72 MPa (−22.8%)
SCWTR	5	31.63 MPa	33.20 MPa (+5.0%)/31.63 MPa (0%)	30.57 MPa (−3.4%)
	10	29.79 MPa	31.21 MPa (+4.8%)/29.78 MPa (≈0%)	28.18 MPa (−5.4%)
	20	21.62 MPa	29.21 MPa (+35.0%)/22.95 MPa (+6.1%)	22.44 MPa (+3.8%)
LCWTR	5	34.98 MPa	35.92 MPa (+2.7%)/36.98 MPa (+5.7%)	22.33 MPa (−36.2%)
	10	31.54 MPa	32.61 MPa (+3.4%)/33.66 MPa (+6.7%)	19.30 MPa (−38.8%)
	20	28.77 MPa	30.13 MPa (+4.7%)/30.41 MPa (+5.7%)	18.14 MPa (−36.9%)

**Table 5:** Two-way ANOVA and multivariate regression results confirming the interaction between WTR type and WTSF ratio on compressive strength.

Source of Variation	df	*F*-Value	*p*-Value	Significance
WTR type	2	19.84	<0.001	***
*WTSF* (%)	3	27.42	<0.001	***
Interaction (WTRXWTSF)	6	4.91	0.0017	**
Residual error	36	-	-	-

Statistical significance levels—*p* < 0.05 (-), *p* < 0.01 (**), *p* < 0.001 (***).

**Table 6:** Failure Modes vs. Strength Relation with Explanations.

Mix ID	WTR Type	WTSF Ratio	Compressive Strength (MPa)	Failure Mode	Observations
0WTR-0WTSF	-	-	34.98	Brittle splitting	Sudden vertical cracks along loading axis; clean fracture surfaces with limited energy absorption; typical of plain concrete.
10FWTR-0WTSF	Fine (10%)	0	25.95	Brittle–weak ITZ	Wide cracks and aggregate–paste separation due to weak rubber–cement interface; low density and high porosity accelerate brittle failure.
5LCWTR-1WTSF	Large (5%)	1%	36.98	Ductile–bridging	Multiple fine cracks with gradual propagation; clear evidence of steel fiber pull-out; Fibers bridge cracks and enhance post-peak load capacity.
20FWTR-2WTSF	Fine (20%)	2%	16.72	Brittle–heterogeneous	Severe segregation, fiber clumping, and a weak matrix, local crushing and block-type collapse, loss of homogeneity that explains the sharp strength reduction.

**Table 7:** The ranking and corresponding mean absolute SHAP values.

Feature	Mean SHAP Value	*RI* (%)	Effect Direction
Bulk density (*p*)	0.091	41.3	Positive—higher density increases strength
*WTR* (%)	0.067	30.4	Negative—higher WTR reduces strength
*WTSF* (%)	0.039	17.7	Positive up to 1%, negative beyond 2%
WTR Type	0.023	10.6	Positive—coarse particles (LCWTR, SCWTR) mitigate losses

## Data Availability

The original contributions presented in the study are included in the article; further inquiries can be directed to the corresponding authors.
